# Ziyuglycoside II ameliorates chemotherapy-induced neutropenia by promoting neutrophil differentiation and functional recovery via SPI1 and C/EBPϵ transcriptional regulation

**DOI:** 10.3389/fimmu.2026.1771161

**Published:** 2026-02-26

**Authors:** Lingdi Li, Huan Lei, Luqi Chen, Chunye Cao, Haolin He, Yanfeng Zhang, Lin Zhang, Liang Peng, Yuxiu Yang, Yulin Feng, Haihong Fang

**Affiliations:** 1 School of Pharmacy, Jiangxi Science and Technology Normal University, Nanchang, China; 2 The National Pharmaceutical Engineering Center for Solid Preparation in Chinese Herbal Medicine, Jiangxi University of Chinese Medicine, Nanchang, China

**Keywords:** C/EBPϵ, chemotherapy-induced neutropenia, neutrophil differentiation, SPI1, transcriptional regulation, Ziyuglycoside II

## Abstract

**Background:**

Chemotherapy-induced neutropenia (CIN) remains a major dose-limiting toxicity associated with myelosuppressive chemotherapy regimens. The development of therapeutic strategies capable of effectively restoring neutrophil production and function could address a critical unmet clinical issue. ZGSII, a bioactive compound derived from *Sanguisorba officinalis*, has shown potential in ameliorating leukopenia. To further evaluate its therapeutic applicability for CIN, a comprehensive understanding of its underlying mechanisms is essential. This study aims to assess the efficacy of ZGSII in mitigating cyclophosphamide-induced neutropenia and myelosuppression and to elucidate the underlying mechanism involved through transcriptome sequencing, protein-protein interaction network construction, and functional validation assays.

**Methods:**

A murine model of cyclophosphamide-induced neutropenia and the human promyelocytic leukemia cell line NB4 were employed to evaluate the effects of ZGSII alleviating chemotherapy-induced neutropenia and promoting neutrophil differentiation, through blood cell count, flow cytometry analysis, and Wright-Giemsa staining. The underlying molecular mechanisms of ZGSII in treatment CIN were systematically investigated through integrated approaches including transcriptomics profiling, computational simulations, and *in vivo* function validation.

**Results:**

This study demonstrated that ZGSII effectively alleviates cyclophosphamide-induced neutropenia and bone marrow suppression in murine models, promoting neutrophil reconstitution without inducing excessive bone marrow mobilization. Transcriptomic analysis revealed that ZGSII restores neutrophil-related transcriptional programs, enriched pathways associated with leukocyte migration, myeloid cell activation, and inflammatory regulation. Integration of publicly granulopoiesis datasets enabled the identification of 37 key genes associated with neutrophil differentiation and maturation. Mechanistically, computational modeling suggests potential interaction of ZGSII with SPI1 and C/EBPϵ, restoring their protein expression and driving granulocytic differentiation. Functional assays further confirmed that ZGSII enhances neutrophil phagocytosis activity, reactive oxygen species (ROS) production, and cytokine homeostasis. Notably, administration of ZGSII conferred significant survival advantages in neutropenic mice following challenge with *Staphylococcus aureus*.

**Conclusions:**

ZGSII alleviates CIN by regulating SPI1 and C/EBPϵ transcriptional activity to promote neutrophil differentiation and functional recovery. These findings provides a preclinical proof for ZGSII as a therapeutic adjuvant or alternative treatment option for CIN.

## 1 Introduction

Chemotherapy-induced neutropenia (CIN), characterized by a reduction in the absolute neutrophil count (ANC) to below 1,500 cells/mm^3^ in peripheral blood, is a common hematological complication associated with myelosuppressive chemotherapy regimens (1). By damaging hematopoietic stem cells and disrupting the bone marrow stromal microenvironment, chemotherapy impairs neutrophil production and maturation, thereby, increasing the risk of severe infections and potentially leading to treatment interruptions (2).

Granulocyte colony-stimulating factors (G-CSFs), which were first introduced clinically in the 1990s, effectively elevate neutrophil counts by activating G-CSF receptors (3). Recombinant G-CSF, as a mainstay prophylaxis agent, reduces the incidence of febrile neutropenia, infections, and antibiotic use, thereby improving post-chemotherapy survival rates (4). However, its clinical utility is limited by a transient duration of action requiring frequent injections, as well as potential adverse effects ranging from bone pain and splenomegaly to more severe complications such as secondary myeloid leukemia and myelodysplastic syndromes (5, 6). Consequently, novel agents that promote sustained hematopoietic reconstitution through intrinsic regulatory mechanisms are urgently needed.

Traditional Chinese medicine (TCM) provides an opportunity to reduce CIN, as it has been found that the therapy has an immunomodulatory effect and low toxicity (7–9). *Sanguisorba officinalis L* has the effects of promoting hemostasis, wound healing, anti-cancer, anti-infection, and improving myelosuppression (10–13). Diyu Shengbai Tablets, prepared from *Sanguisorba officinalis L*, have been widely used for the relief or as an adjuvant therapy for chemotherapy- or radiotherapy-induced myelosuppression and leukopenia (14–16). Ziyuglycoside II (ZGSII), a potent bioactive compound extract from *Sanguisorba officinalis L* and an *in vivo* metabolite of Zyglycoside I (ZGSI), has demonstrated diverse anti-cancer, antioxidant and immunomodulatory effects in preliminary studies (17–19). Pharmacokinetic studies demonstrated that subcutaneous administered prolonged the exposure duration of ZGS I in leukopenia-induced mice compared to intravenous administration and increased the area under the concentration-time curve (AUC_0–t_) of ZGSII (20). Our prior study had established that ZGSII exerts a significant alleviating effect on cyclophosphamide-induced leukopenia in mice, as evidenced by increased peripheral blood cell and neutrophil counts, as well as restoration of bone marrow stem and progenitor cell populations (21). Although these results are encouraging, the mechanisms by which ZGSII regulates neutrophil differentiation and functional activation remain unclear, representing a crucial gap in current knowledge. Despite evidence indicating that ZGSII contributes to the alleviation of chemotherapy-induced neutropenia, the specific molecular mechanisms have yet to be fully elucidated.

Therefore, we integrated *in vivo* and *in vitro* experiments with transcriptomic profiling, protein interaction analysis, and functional validation to elucidate the mechanisms by which ZGSII ameliorates chemotherapy-induced neutropenia. The results demonstrate that ZGSII reversed cyclophosphamide-induced neutropenia and transcriptional dysregulation, promoted neutrophil differentiation, enhanced neutrophil phagocytosis and reactive oxygen species production, and improved survival in neutropenic infection models. This study aims to provide preclinical evidence supporting the therapeutic potential of ZGSII for leukopenia and neutropenia.

## 2 Materials and methods

### 2.1 Chemicals and reagents

Ziyuglycoside II (purity > 98%) was obtained from the National Pharmaceutical Engineering Center for Solid Preparation in Chinese Herbal Medicine (Nanchang, China). A stock solution (100 mg/mL) was prepared in sterile 0.9% (w/v) sodium chloride (NaCl). Cyclophosphamide (Baxter Oncology GmbH, Frankfurt, Germany) was diluted in 0.9% NaCl for *in vivo* administration. Recombinant human granulocyte colony-stimulating factor (rhG-CSF) was purchased from Amoytop Biotech Co., Ltd. (Xiamen, China). The antibodies used for flow cytometer were purchased from eBioscience (Thermo Fisher, USA). The primary antibodies used in Western blot were purchased from Proteintech (Wuhan, China).

### 2.2 Animals and drug administration

Specific pathogen-free (SPF) C57Bl/6J mice (male; 6–8 weeks old; 18 ± 2 g) were obtained from Beijing Kerisi Company Biotechnology Co., Ltd. (License: SCXK(Jing) 2019-0010). Mice were housed under controlled conditions (22 ± 1 °C, 55 ± 5% humidity, 12 hr light/dark cycle). All animal experiments were conducted in the AAALAC-accredited facility of Jiangxi Science and Technology Normal University (IACUC Approval No. Y202340).

After one week of acclimatization, the mice were randomly assigned into five groups: Vehicle control group (Control, 0.9% saline), cyclophosphamide model group (CY): single CY dose; positive control group (G-CSF): CY + rhG-CSF (40 μg/kg/day); ZGSII group (ZGSII): CY + ZGSII (12 mg/kg/day), as our previous research (21). All mice were injected intraperitoneally with cyclophosphamide (CY; 150 mg/kg) in 0.9% NaCl on Day 0, except for the Control group, which received the same volume of saline solution. The administration groups were injected with subcutaneous rhG-CSF for days 1–4 or ZGSII for days 1-5, as described in our previous study. The body weights of mice were recorded daily (± 0.1 g).

### 2.3 Peripheral blood counts, serum biochemical levels, and cytokine measurement

At the designated time point for the experiment, the peripheral blood of mice was collected from the retro-orbital plexus under isoflurane anesthesia. Hematological analysis was performed using BC-5000Vet veterinary hematology analyzer (Mindray, Shenzhen, China), which quantified leukocyte differentials (neutrophils, lymphocytes, monocytes) and erythrocyte parameters.

Blood samples were collected from the retro-orbital plexus and centrifuged at 3,000 g for 15 min; the supernatant serum was aliquoted and stored at -80 °C until analysis. The serum levels of alanine aminotransferase (ALT), aspartate aminotransferase (AST), total protein (TP), albumin (ALB), urea (UREA), and creatinine (CREA) were determined using an automated clinical chemistry analyzer (Chemray240, Shenzhen) according to the manufacturer’s instructions. Commercially available reagent kits with calibrated standards were used for each parameter to ensure accuracy. The serum concentrations of GM-CSF, IL-6, and TNF-α were quantified using commercial enzyme-linked immunosorbent assay (ELISA) kits (Baipeng Biotechnology Co., Ltd., China) following the provided instructions. A standard curve was generated for each assay, and cytokine concentrations for unknown samples were calculated by interpolation. All samples and standards were assayed in duplicate.

### 2.4 Hematoxylin-eosin staining

Femurs were dissected, decalcified in EDTA, dehydrated through an ethanol series, cleared in xylene, and paraffin-embedded. Longitudinal sections (4 μm) were stained using hematoxylin and eosin (H&E). Representative images were acquired at 100× and 400× magnification using bright-field microscopy.

### 2.5 Organ index assessment

The thymus and spleen were excised, blotted dry, and weighed. Organ indices were calculated as (Organ index (mg/g) = Organ weight (mg)/Body weight (g).

### 2.6 Cell culture

The human acute promyelocytic NB4 cell line was acquired from Baidi Biotech Co., Ltd. (Baidi Biotech, Shanghai), and authenticated using short tandem repeat (STR) profiling. Cells were cultured in RPMI-1640 medium (Servicebio, Wuhan) supplemented with 10% FBS and 1% penicillin-streptomycin at 37 °C/5% CO_2_.

### 2.7 Cell viability

Cell viability was assessed using the Cell Counting Kit-8 kit (CCK-8; Biosharp Life Sciences, Hefei). Cells were seeded in 96-well plates at a density of 5 × l0^3^ cells/well and allowed to adapt for 4–6 hr under standard culture conditions. After treatment with serially diluted concentrations of ZGSII (24–120 hr, 24-hour intervals), 20 μL of CCK-8 reagent was added to each well and incubated for 4 hours at 37 °C. The optical density (OD) was measured at 450 nm using a multimode microplate reader (SpectraMax Id3, California, USA).

### 2.8 Flow cytometry analysis of cell differentiation and maturation

*In vivo neutrophil maturation:* Bone marrow cells from CIN mice were lysed using ACK lysing buffer with 10 min incubation at 25 °C. Cells were stained with CD11b-FITC (1:100; 11-0112-82), and Ly-6G/Ly6C-Alexa Fluor 700 (1:100; 56-5931-82) (eBioscience). Antibody-cell mixtures were incubated for 30 min at 4 °C in the dark, followed by two PBS washing cycles (400 ×g, 5 min). Cells were resuspended in 300 μL PBS and analyzed using a Gallios flow cytometer (Beckman Coulter). Neutrophil subpopulations were quantified through Ly-6G^+^CD11b^+^ dual-positive gating strategies (22), and mature neutrophils (CD11b^+^ Ly6G^+high^), immature neutrophils (CD11b^+^ Ly6G^+low^) (22, 23) were quantified using Flowjo.

*In vitro differentiation:* NB4 cells in the logarithmic growth phase were seeded into 6-well plates at 5 × 10^4^ cells/mL (2 mL/well) and were treated with a serial concentration of ZGSII. After 120 hours of incubation, cells were harvested by centrifugation (2000 rpm, for 5 min, 4 °C) and washed twice with ice-cold PBS. Surface marker staining was conducted with fluorochrome-conjugated antibodies CD11b-PE (1:100; 12-0118-42).

### 2.9 Wright-Giemsa staining

*For Peripheral blood staining:* Peripheral blood (5 μL) was smeared, air-dried, and stained with Swiss-Giemsa solution. The morphology of neutrophils was examined and calculated.

*In vitro cell staining*: NB4 cells (1×10^6^/mL) were smeared, air-dried, fixed in 4% paraformaldehyde (PFA), and stained (Solution A/B, Servicebio). After drying, morphology was captured using a BDS400 inverted microscope (Leica Microsystems, Wetzlar, Germany), and digital images were acquired at 100 × magnification for quantitative analysis.

### 2.10 Flow cytometry analysis of cell cycle and apoptosis

NB4 cells were seeded into 6-well plates at 1 × 10^5^ cells/mL and were treated with a serial concentration of ZGSII. After 120 hours of incubation, cells were harvested and the cell cycle distribution and the apoptosis rate was detected by the cell cycle kit (KGA512, KeyGen Biotech, China) and the Annexin V-APC/PI Apoptosis Detection Kit (KGA1030, KeyGen Biotech, China). For cell cycle analysis, harvested cells were washed in PBS and fixed with ice-cold 70% ethanol solution for overnight. After washed, the PI/RNase A (9:1) working solution was added in accordance with the proportion of cells. The cell cycle distribution was determined by a BD flow cytometer and analyzed with the Flowjo (version 10) software. For apoptosis analysis, harvested cells were washed with PBS, resuspended in binding buffer, and incubated with Annexin V-FITC/PI fluorescent dyes. The apoptosis rate of each sample was detected by flow cytometer and analyzed with the Flowjo (version 10) software.

### 2.11 Bacterial phagocytosis and survival assays

Gram-positive Staphylococcus aureus *(Staphylococcus aureus, S. aureus*, ATCC6538) was used for *in vitro* phagocytosis and survival rate assay.

*Phagocytosis:* Peripheral blood (10 μL) was incubated with an equal volume of log-phase *S. aureus* at 37 °C for 30 minutes. Following incubation, blood-bacterial smears were stained with the Swiss-Giemsa solution. A total of one hundred neutrophils were randomly counted, and the percentage of phagocytosing neutrophils was enumerated microscopically.

*Survival rate*: CIN mice were challenged i.p. with approximately 5 × 10^7^ CFU of S. aureus on Day 4. Mouse mortality was recorded every 12–24 h or 10 days to assess short-term and long-term survival rates. The Log-rank test was used to compare the median survival rate or actual survival rate of mice.

### 2.12 NBT reduction assay

Peripheral blood samples were incubated with PBS containing PMA (1 μg/mL) and NBT (1 mg/mL) for 45 min. Following incubation, blood smears were Giemsa-stained. A total of one hundred neutrophils were randomly counted, and the proportion of NBT-positive neutrophils (those containing formazan particles) was calculated.

### 2.13 Bioinformatics analysis

#### 2.13.1 Transcriptome RNA sequencing

Bone marrow cells of 6–8 mice each group were isolated on days 4 and 6 post-CY administration. Total RNA was extracted by TRIzol™ Reagent (Thermo Fisher Scientific, 15596026), with integrity verified (RIN > 8.0; DV200 > 85%) using a Fragment Analyzer System. Sequencing libraries were prepared using the MGISEQ-2000 platform specifications (MGI Tech, Wuhan, China). Following Illumina library construction, raw reads underwent quality control via SOAPnuke v2.1.5 (24), and clean reads were aligned to the reference genome sequence using Bowtie2 (v2.4.5) (25). Gene expression quantification was performed with RSEM (v1.3.1) (26) Differential expression analysis was conducted using DESeq2and DEGSeq (27), with significance thresholds set at |log2 (fold change)| >1 and FDR-adjusted P <0.05. DEGs of all samples are provided in **Supplementary Table 1**.

#### 2.13.2 Immune infiltration and time-series trajectory analysis

Differential expression genes (DEGs) identified from RNA-seq data were analyzed using CIBERSORT to quantify the relative proportions of 25 predefined immune cell subtypes across sample groups (28, 29). Immune cell composition was visualized using stacked bar plots. Subsequent time-series trajectory analysis (days 4-6) of DEGs was performed to characterize temporal expression patterns, revealing genes that reversed CY-induced alterations. The heatmap was plotted by an online platform for data analysis and visualization (https://www.bioinformatics.com.cn). C1 and C3 cluster DEGs are provided in **Supplementary Table 2**.

#### 2.13.3 Functional enrichment analysis

Functional enrichment analysis of Gene Ontology (GO) terms and Kyoto Encyclopedia of Genes and Genomes (KEGG) pathways was conducted on the intersecting genes. Enriched categories were identified using a hypergeometric test with a false discovery rate (FDR)-adjusted p-value < 0.05.

#### 2.13.4 Neutrophil-related genes acquisition

The GSE137539 dataset was retrieved from the GEO database (https://www.ncbi.nlm.nih.gov/geo). To identify the neutrophil-associated genes, four normal mouse samples in the dataset were selected for further analysis. The data were loaded and integrated into the R package Seurat, and the top 2,000 highly variable genes were identified. For downstream clustering, a nearest-neighbor graph was constructed based on the first 5 corrected PCs, followed by cell clustering with FindClusters at a resolution of 0.5. The resulting clusters were visualized using UMAP. All cells were annotated by leveraging the cell type and G0-G5 phase (neutrophil subpopulations) designations (30).

#### 2.13.5 Neutrophil subpopulation pseudotemporal analysis

The neutrophil subset was isolated for further analysis, and G0-G5 clustering was performed using the first 10 PCs with FindNeighbors and FindClusters (resolution = 0.5). Subsequently, cellular stemness was evaluated with CytoTRACE2, and the cluster exhibiting the highest score was defined as the developmental origin. The developmental trajectory was inferred, and genes that varied significantly along pseudotime were computed using Monocle3.

#### 2.13.6 Protein-protein interaction network construct

Finally, a protein-protein interaction (PPI) network for the key genes was constructed using the STRING database, with a minimum required interaction confidence score of 0.4.

#### 2.13.7 Transcription factors prediction

To identify transcription factors (TFs) enriched for the key genes, the gene set was analyzed using the CHEA3 tool. The top 10 ranked TFs were selected for further investigation. Subsequently, a TF-gene regulatory network was constructed based on the interaction relationships among the key genes.

### 2.14 Molecular docking studies

Molecular docking simulations between ZGSII and key TFs were performed using AutoDock vina 1.2.3 (31). The crystal structure of SPI1 (PDB ID: 8e3k) was acquired from the PDB, while the C/EBPϵ model was sourced from AlphaFold. The 3D structure of ZGSII was obtained from PubChem and energy-minimized under the MMFF94 force field. Before docking, protein receptors were processed with PyMOL 2.5.2 to remove heteroatoms. The conformation yielding the highest binding score was designated as the putative binding mode and visualized in PyMOL 2.5.2.

### 2.15 Molecular dynamics simulations

Molecular dynamics simulations were performed in AMBER 24 (32), using the highest-ranked docking pose as the initial structure. The protein-ligand complex was solvated in a TIP3P water box (10 Å cutoff), neutralized with ions, and the necessary topology files were generated. The simulation protocol began with energy minimization, a stepwise equilibration phase, solvent relaxation, and system density equilibration for 500 ps at 298.15 K and 1 atm (NPT). Calculations used a 10 Å cutoff, PME for electrostatics, the SHAKE algorithm, a Langevin thermostat (γ = 2 ps^-^¹), and a 2 fs timestep, with coordinates recorded every 20 ps. After the simulation was completed, trajectories were analyzed for root mean square deviation (RMSD), root mean square fluctuation (RMSF), radius of gyration (Rg), solvent accessible surface area (SASA), and hydrogen bond formation (HBond). The binding free energy (MM/GBSA) was calculated (33).

### 2.16 Quantitative real-time polymerase chain reaction

Gene expression in bone marrow cells was quantified via mRNA analysis. Total RNA was extracted using TRIzol reagent, and then reverse-transcribed into cDNA with HiScript II Q RT SuperMix (Vazyme Biotech). Quantitative PCR analysis was performed using ChamQ Universal SYBR qPCR Master Mix on a CFX Duet Real-Time System (BIO-RAD, USA), with gene expression levels normalized to 18S rRNA and calculated with the 2^-ΔΔCT^ method. Primer sequences are provided in **Supplementary Table 3**.

### 2.17 Western blot analysis

Bone marrow cells were collected and lysed with RIPA buffer. The protein concentration was quantified using a BSA assay kit (Solarbio, China). The protein samples were separated by 10% SDS-PAGE gels and transferred onto PVDF membranes. The membranes were blocked with 5% skim milk in TBST for 1.5 h at room temperature. The primary antibodies of LTF (10933-1, 1:5000), CXCR2 (85144-5,1:2000), SPI1 (66618-2,1:2000), C/EBPϵ (14271-1, 1:800), and β-actin (1:100000) were incubated overnight at 4 °C. The HRP-conjugated secondary antibodies were incubated at room temperature for 1 h at room temperature. Bands were visualized using an imaging system (Biorad, USA) and quantified with ImageJ software.

### 2.18 Statistical analysis

All experimental data were presented as the mean ± standard deviation (SD). Each independent experiment was replicated a minimum of three times to determine the mean, and the number of experiments in each group satisfied the statistical requirements. Statistical comparisons between experimental groups were performed using one-way ANOVA or student’s t-tests (GraphPad Prism 8.0). A pre-study sample size calculation was performed to ensure adequate statistical power. A p-value of less than 0.05 (*P* < 0.05) was considered statistically significant, indicating a meaningful difference between groups.

## 3 Results

### 3.1 ZGSII mitigates cyclophosphamide-induced neutropenia and bone marrow suppression

**Figure 1:**
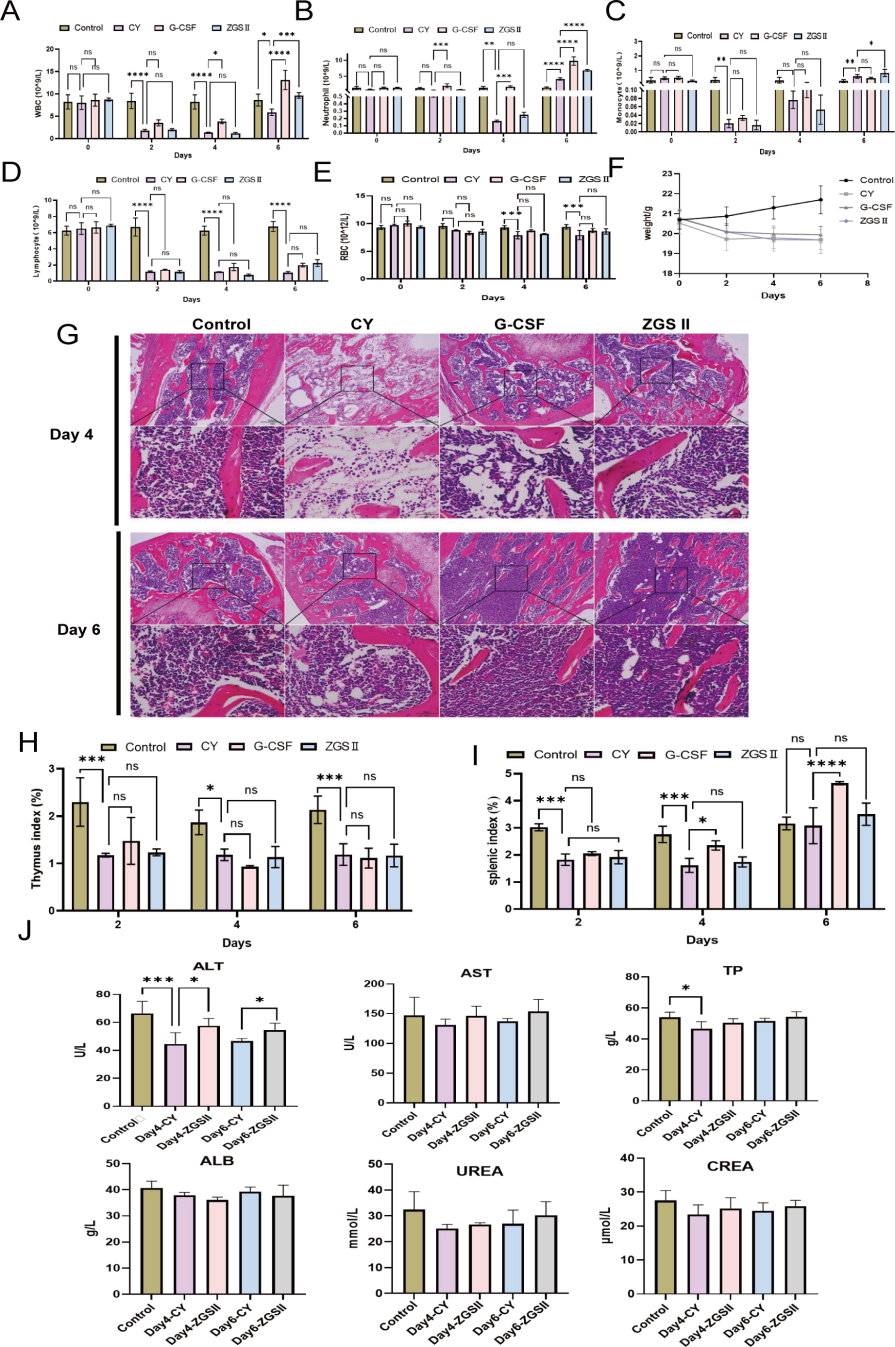
ZGSII alleviates CIN and bone marrow suppression. **(A-E)** Peripheral blood cell counts in CIN mice: **(A)** white blood cells (WBC), **(B)** neutrophils, **(C)** monocytes, **(D)** lymphocytes, and **(E)** red blood cells (RBC). **(F)** Body weight. **(G)** Representative hematoxylin and eosin (H&E)-stained femoral bone marrow at 100 × (upper panels) and 400 × (lower panels) magnification on days 4 and 6 CY post-induction (scale bar: 100 μm). **(H)** The thymus index and **(I)** the splenic index were calculated as organ-to-body weight ratios. **(J)** The serum biochemical level, including ALT, AST, TP, ALB, UREA, and CREA. Data represent mean ± SD (n = 6–8). Statistical significance versus CY model group: *P < 0.05, **P < 0.01, ***P < 0.001, ****P < 0.0001.

To evaluate the therapeutic efficacy of ZGSII in treating chemotherapy-induced neutropenia (CIN), mice were administered ZGSII (12 mg/kg/day for 5 days) or rhG-CSF (40 μg/kg/d for 4 days) as a positive control following cyclophosphamide (CY) injection. Peripheral blood cell counts, including white blood cells (WBCs), neutrophils, monocytes, and lymphocytes, and red blood cells (RBCs) were measured on days 0, 2, 4, and 6 post-CY treatment using an automated hematology analyzer. As expected, CY administration resulted in a marked reduction in leukocyte counts by days 2 and 4, confirming successful establishment of leukopenia and neutropenia (**Figures 1A–D**). Body weight significantly decreased following CY administration (**Figure 1F**).

G-CSF treatment significantly increased WBC and neutrophil levels from day 4 onward. In contrast, ZGSII induced a more gradual recovery, ultimately elevating WBC (↑1.6-fold, *P* < 0.001) and neutrophil counts (↑1.7-fold, *P* < 0.001) by day 6 compared to the CY group (**Figures 1A, B**). G-CSF produced a more remarkable increase in WBC (↑2.2-fold) and neutrophils (↑2.4-fold). ZGSII also promoted a progressive recovery in monocyte counts (**Figure 1C**), whereas neither treatment significantly affected lymphocyte or RBC levels (**Figures 1D, E**). Concurrent with the development of neutropenia, a significant decrease in body weight was observed following CY challenge (**Figure 1F**).

Histological analysis of H&E-stained bone marrow sections revealed severe hypocellularity in CY-treated mice at day 4, with partial recovery observed by day 6. Both ZGSII and G-CSF significantly improved marrow cellularity at these time points (**Figure 1G**). While ZGSII did not affect thymic or splenic indices, G-CSF induced marked splenomegaly (**Figures 1H, I**).

To evaluate potential hepatorenal toxicity, serum biochemical parameters were analyzed (**Figure 1J**). CY treatment significantly reduced ALT and total protein (TP) levels. ZGSII restored ALT levels, and TP level returned to baseline by day 6. No significant changes were observed in AST, ALB, urea, and creatinine (CREA) levels.

### 3.2 ZGSII promotes neutrophil reconstitution without excessive marrow mobilization

**Figure 2:**
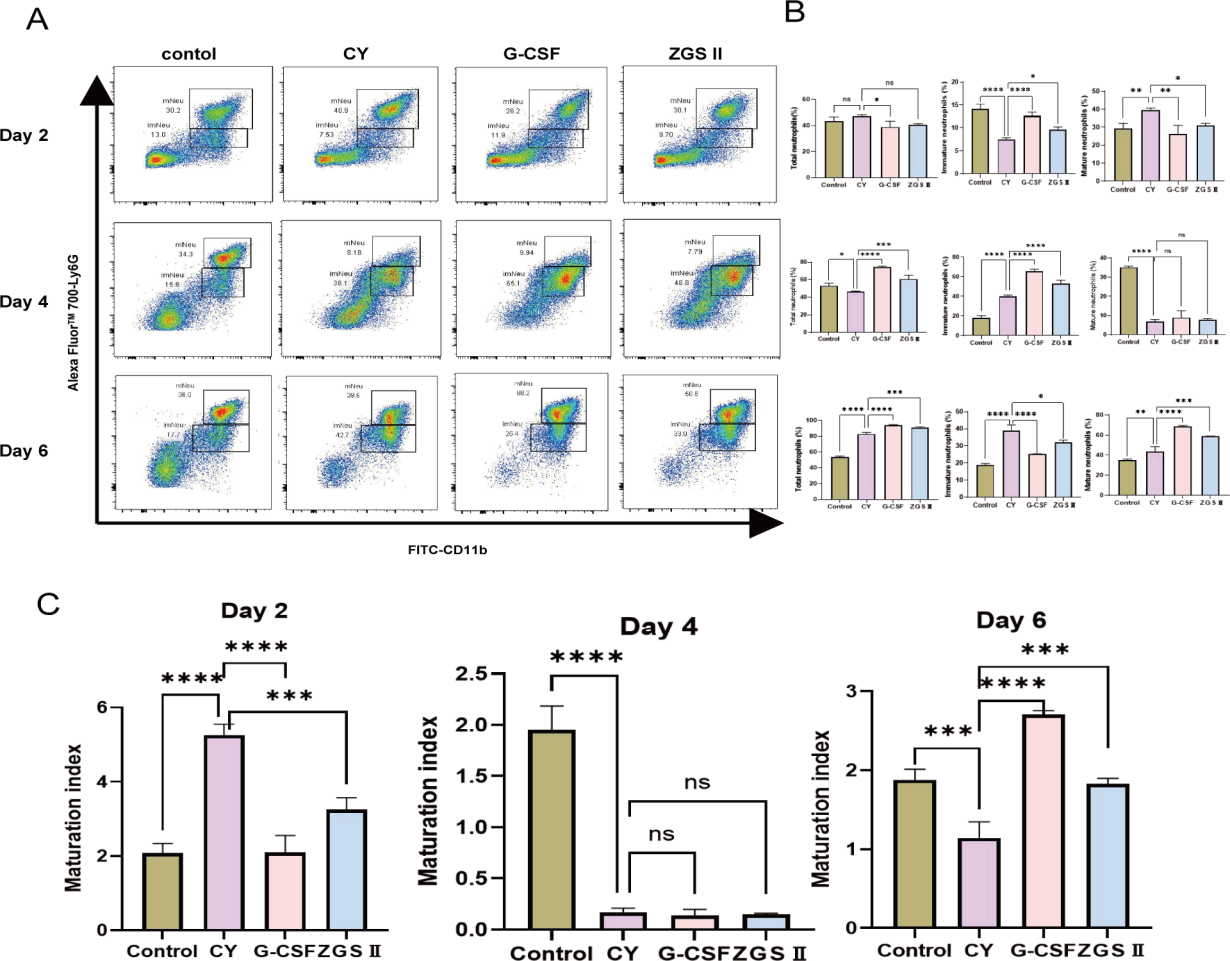
Ziyuglycoside II enhances neutrophil maturation in CIN mice. **(A)** Representative flow cytometry plots. **(B)** Quantitative analysis of bone marrow neutrophil subpopulations: total neutrophils (CD11b^+^Ly6G^+^), mature neutrophils (CD11b^+^ Ly6G^+high^), and immature neutrophils (CD11b^+^ Ly6G^+low^). **(C)** Maturation index (mature: immature neutrophil ratio). Data represent mean ± SD (n=6-8). *P < 0.05, **P < 0.01, ***P < 0.001, ****P < 0.0001 versus CY group.

To investigate the impact of ZGSII on neutrophil development, bone marrow cells were collected from CIN mice on days 2, 4, and 6 post-treatment. Flow cytometric analysis was performed to quantify neutrophil subpopulations: total (CD11b^+^Ly6G^+^), mature (CD11b^+^Ly6G^+ high^), and immature (CD11b^+^Ly6G^+ low^) neutrophils. Within two days post-induction, CY treatment significantly decreased the proportions of immature neutrophils, while increasing that of mature neutrophils (*P* < 0.001 vs. control), suggesting that CY-induced myelosuppression may concurrently stimulate emergency granulopoiesis (**Figures 2A, B**). From day 2 to day 4, G-CSF significantly expanded the immature neutrophil pool, whereas ZGSII induced a more moderate response.

By day 6, total neutrophil levels in both treatment groups surpassed baseline values (1.5-fold increase, *P* < 0.01**).** Although CY-treated mice maintained an elevated immature neutrophil fraction (38.3% vs. 18.7% in controls), G-CSF was more effective than ZGSII in increasing mature neutrophils (1.58-fold vs. 1.3-fold increase relative to CY, *P* < 0.001) (**Figures 2A, B**). The neutrophil maturation index (mature/immature ratio) decreased sharply in CY-treated mice by day 4 (11.4-fold reduction vs. control, *P* < 0.001). By day 6, this ratio was restored to near-normal levels by ZGSII (97% of control) and above-normal by G-CSF (144% of control) (**Figure 2C**). These results demonstrate that while both agents promote neutrophil production, G-CSF may excessively drive bone marrow mobilization, whereas ZGSII supports a more balanced reconstitution without overt depletion of hematopoietic reserves.

### 3.3 ZGSII reverses CIN by restoring transcriptional programs and promoting neutrophil maturation

**Figure 3:**
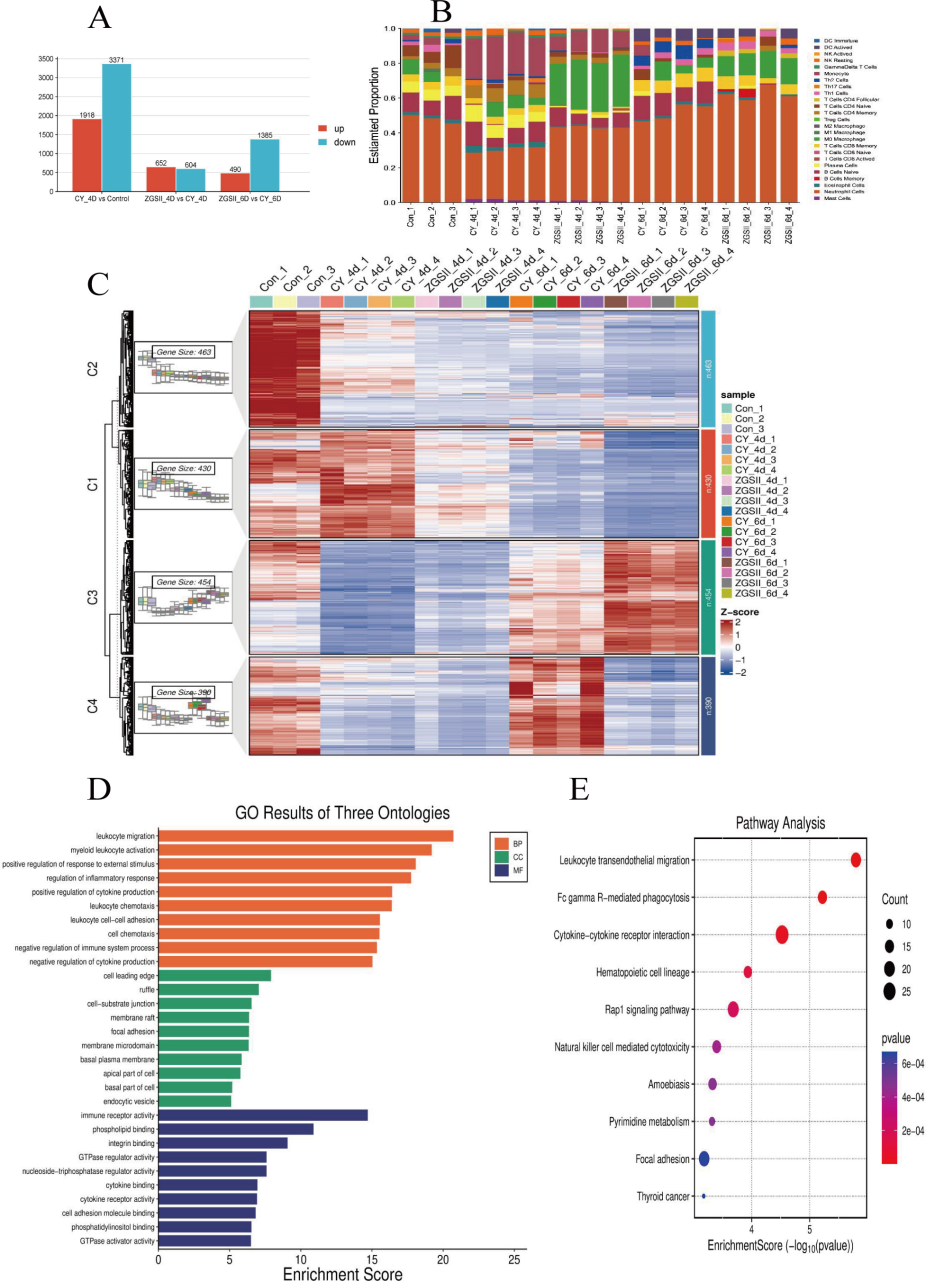
Transcriptomic profiling reveals ZGSII-mediated restoration of neutrophil homeostasis. **(A)** Differentially expressed genes (DEGs) across experimental groups (threshold: |log_2_FC| >1, FDR-adjusted *P* < 0.05). **(B)** Immune cell infiltration landscape in bone marrow quantified by CIBERSORT analysis. **(C)** Heatmap of temporally expressed genes (Days 4-6) identified through time-series clustering. Rows: Z-score normalized expression; columns: individual samples. **(D)** Gene Ontology biological processes (BP), cellular component (CC), and molecular function (MF). **(E)** KEGG signaling pathways. Dot size represents gene count; color intensity indicates -log_10_ (FDR-adjusted *P*-value).

The aforementioned findings that the white blood cell and neutrophil counts reached their nadir on day 4, suggesting the phase of cyclophosphamide-induced bone marrow suppression. Subsequently, a recovery in white blood cells and neutrophils was observed, with a statistically significant difference emerging between the ZGSII-treated group and the model group on day 6. To elucidate the mechanisms by which ZGSII promotes neutrophil differentiation and ameliorates neutropenia, we performed RNA sequencing (RNA-seq) on bone marrow samples collected at days 4 and 6 CY post-induction. Comparative transcriptomic analysis between CY-treated and control mice revealed 5,289 differentially expressed genes (DEGs), including 3,371 downregulated and 1,918 upregulated genes (**Figure 3A**). In ZGSII-treated mice compared to CY controls, 1,256 DEGs were identified at day 4 (604 downregulated, 652 upregulated). By day 6, ZGSII treatment resulted in 1,875 DEGs relative to CY controls (1,385 downregulated; 490 upregulated; **Figure 3A**).

We then profiled immune cell infiltration to evaluate changes in the bone marrow microenvironment. The relative abundance of 25 murine immune cell subsets was determined using the CIBERSORT algorithm. CY-treatment also resulted in the robust depletion of bone marrow neutrophils on day 4 that was reversed by ZGSII. By day 6, the neutrophil levels were once again increased (**Figure 3B**).

Subsequently, the transcriptomic data were subjected to time-series clustering, which revealed four distinct expression patterns. In cluster 1 (C1) and cluster 3 (C3), ZGSII was observed to cause significantly reverse the transcriptional alterations induced by CY, whether upregulated or downregulated (**Figure 3C**). Gene Ontology (GO) enrichment analysis indicated that the genes within clusters C1 and C3 were highly enriched in biological processes (BP) such as leukocyte migration, myeloid leukocyte activation, and regulation of inflammatory response, as well as molecular functions (MF) including immune receptor activity and integrin binding (adjusted p < 0.05; **Figure 3D**). Furthermore, KEGG pathway analysis revealed significant enrichment in pathways related to leukocyte transendothelial migration, cytokine-cytotokine receptor interaction, and hematopoietic cell lineage pathway (**Figure 3E**).

### 3.4 ZGSII promotes late-stage neutrophil differentiation and activation via upregulation of key developmental genes

**Figure 4:**
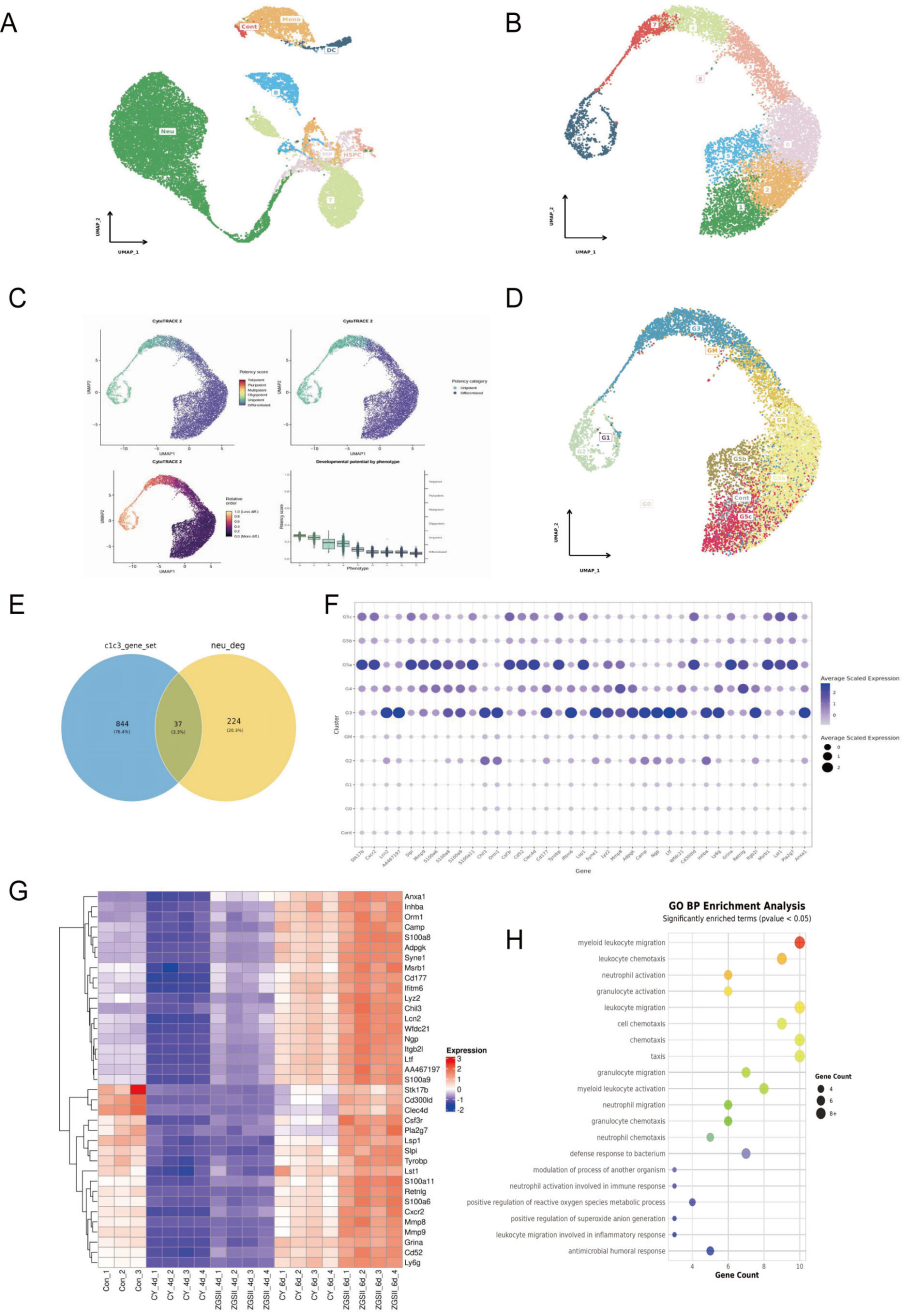
The effects of ZGSII on neutrophil subpopulations. **(A)** Uniform main fold approximation and projection (UMAP) of 19,582 cells from GSE137539. **(B)** The UMAP plot displayed the distribution of Seurat clusters for the high-quality neutrophil-related cells in GSE13753. **(C)** Assessment of stemness neutrophil subsets. **(D)** The pseudotime trajectory plot depicted the neutrophil differentiation and maturation (G0-G5) from left to right. **(E)** Venn diagram of the intersection between the C1C3 gene set and the G0-G5 neutrophil population genes. **(F)** Dot plot showing the transcription gradient of intersection genes from G0-G5. **(G)** The heatmap presented the 37 intersection DEGs expressed in different groups. **(H)** The BP of GO enrichment analysis for 37 intersection DEGs.

To further delineate the granulocytic developmental stages modulated by ZGSII, we analyzed the public dataset GSE137539 from GEO, focusing on four normal mouse samples. Following feature extraction, dimensionality reduction, and clustering, we annotated neutrophil-related populations (myeloid progenitors and neutrophils) based on established markers (30) (**Figure 4A**). Neutrophils were subsequently extracted and reclustered into nine distinct clusters and subpopulations (**Figure 4B**).

Cellular stemness was assessed using the CytoTRACE2 algorithm, which identified that cluster 6 exhibited the highest stemness score and was thus designated as the root for pseudotime trajectory analysis. Genes showing dynamic expression along the inferred trajectory were identified through Moran’s I statistic (Moran’s I > 0.3, p < 0.05, q < 0.05) (**Figure 4C**). Integration of cellular stemness levels with neutrophil developmental stages enabled annotation of the nine clusters into eight distinct neutrophil subpopulations G1-G4, GM, G5a-G5c, except for G0, which no significant enrichment was observed in the 9 clusters (30). Stage-specific enrichment analysis demonstrated that G1 and G2 stages were predominantly represented in cluster 6; G3 in clusters 4 and 7; GM in clusters 8; G4 in clusters 0; G5a-G5c in clusters 2, 1 and 5, respectively (**Figure 4D**).

By intersecting the gene sets of neutrophil subpopulations with the previously defined DEGs in clusters C1 and C3, we identified 37 key genes (**Figure 4E**). These genes were predominantly upregulated following ZGSII treatment and enriched in the G3 to G5 subpopulations, which displayed higher differentiation and maturity scores (**Figures 4F, G**). GO enrichment analysis revealed that these key genes were significantly enriched in biological processes such as myeloid leukocyte migration, leukocyte chemotaxis, and neutrophil activation (**Figure 4H**).

### 3.5 ZGSII promotes granulocyte differentiation and maturation Via transcriptional regulators SPI1 and C/EBPϵ

**Figure 5:**
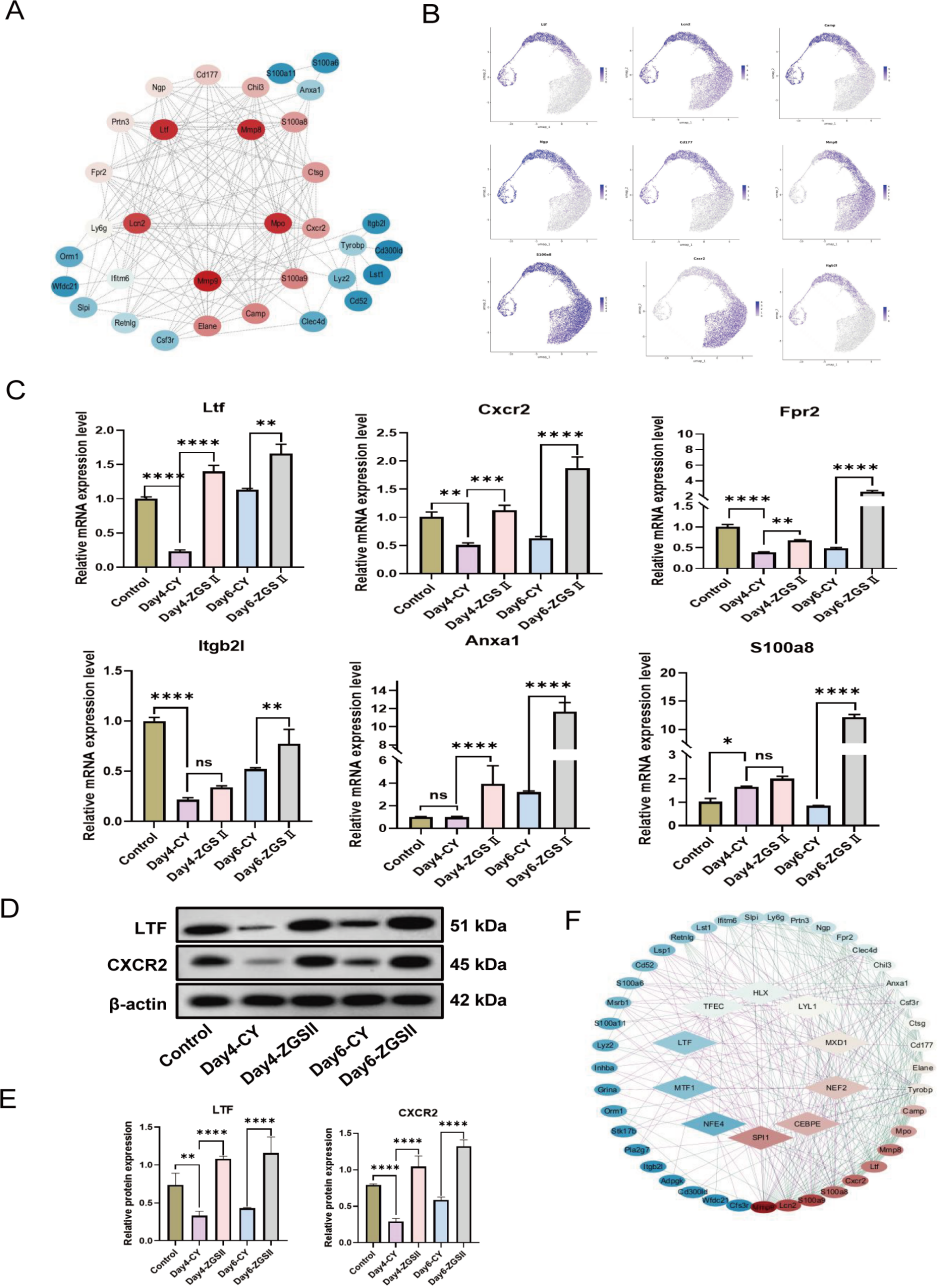
ZGSII regulates core genes and transcription factors. **(A)** PPI network of 37 intersection DEGs. **(B)** The pseudotime trajector plot of *Ltf, Lcn2, Camp, Ngp, Cd177, Mmp8, S100a8, Cxcr2, and Itgb2l***(C)** RT-qPCR validation of key transcripts in bone marrow cells: *Cxcr2*, *Fpr2*, *Itgam*, *Itgb2*, *Anxa1*, and *S100a8*. Data normalized to 18S rRNA. **(D)** Representative Western blot images of LTF and CXCR2 in the BM cells from Con, CY, and ZGSII groups. **(E)** Quantitative analysis of LTF and CXCR2 expression (n = 3). **(F)** The circle plot displayed the crosstalk network between TFs and genes. (mean ± SD, n=3 biological replicates). *P < 0.05, **P < 0.01, ***P < 0.001, ****P < 0.0001 vs. CY model group.

To further elucidate the molecular mechanisms by which ZGSII alleviates CIN, we sought to identify its target genes and associated transcriptional regulators. A protein-protein interaction (PPI) network was constructed based on the 37 key genes using the STRING database (confidence threshold > 0.4), which revealed several hub genes, including *Ltf, Lcn2, Cxcr2, Mmp8/9, and S100a8/9* (**Figure 5A**). Pseudotime trajectory analysis demonstrated stage specific expression patterns during neutrophil maturation: secondary granule genes such as Ltf*, Lcn2, Ngp, and Camp* were predominantly expressed in G1-G3 populations, *Cxcr2* and *Mmp8* were enriched in the highly mature G4-G5 subsets, and *S100a8* was broadly expressed across all stages (G1-G5c) (**Figure 5B**). The mRNA expression levels of selected genes, including *Ltf*, *Cxcr2, Fpr2, Itgb2l, Anxa1*, and *S100a8* were validated by RT-qPCR (**Figure 5C**). Protein expression of LTF and CXCR2 was further confirmed by Western blot analysis (**Figures 5D, E**). The results demonstrate that ZGSII treatment reversed the dysregulation of LTF and CXCR2 at both the transcriptional and translational levels.

Transcription factors (TFs) play a crucial role in regulating the differentiation and maturation of granulocytes. To identify upstream transcription regulators, we analyzed the set of 37 key genes using the CHEA3 platform. Based on the top ten ranked TFs, a TF-gene regulatory network was constructed by integrating the PPI data, which identified SPI1 and C/EBPϵ as the most central regulators (**Figure 5F**). Collectively, these findings indicate that ZGSII promotes neutrophil development and maturation, likely through modulation of SPI1 and C/EBPϵ activity.

### 3.6 Computational simulations reveal binding modes and affinities of ZGSII with SPI1 and C/EBPϵ

**Figure 6:**
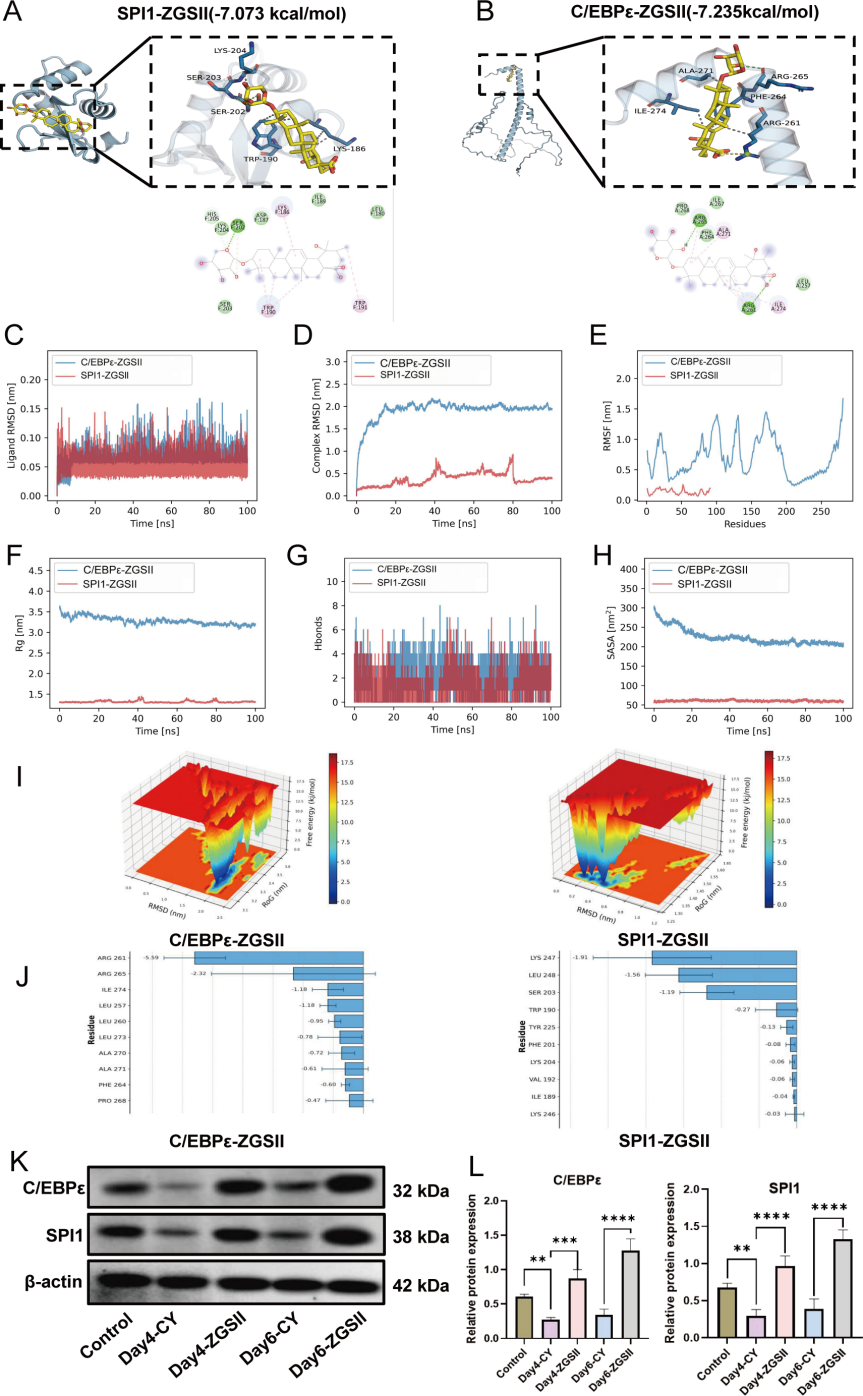
Molecular interactions between ZGSII and transcriptional regulator, and protein validation. **(A, B)** Predicted binding conformations of ZGSII with transcriptional regulators: SPI1 and C/EBPϵ. **(C-H)** Molecular dynamics trajectory analyses of ZGSII-protein complexes over 100 ns: **(C)** Ligand RMSD, **(D)** Complex RMSD. **(E)** Protein RMSF, **(F)** Complex Rg. **(G)** Number of H-bonds, **(H)** Complex SASA. **(I)** Free energy landscape of a complex. **(J)** Contribution of key amino acid binding energy. **(K)** Representative western blot images of SPI1 and C/EBPϵ in the BM cells from Con, CY, and ZGSII groups. **(L)** Quantitative analysis of SPI1 and C/EBPϵ expression (n = 3). **P < 0.01, ***P < 0.001, ****P < 0.0001.

To further elucidate the mechanism of action of ZGSII specifically, the potential interaction of ZGSII with the two transcription factors SPI1 and C/EBPϵ, molecular docking experiments were conducted in this study. The docking results demonstrated that ZGSII exhibits high binding affinity toward both SPI1 and C/EBPϵ, with binding free energies of -7.073 kcal/mol and -7.235 kcal/mol, respectively. In the SPI1-ZGSII complex, ZGSII forms extensive hydrophobic interactions with TRP-190, LYS-204 and LYS-186, contributing to complex stabilization in a nonpolar environment. Additionally, hydrogen bonds are formed between ZGSII and SER-202 and SER-203, which enhance both binding affinity and specificity (**Figure 6A**). The ligand formed hydrophobic interactions within the C/EBPϵ pocket, specifically with residues ALA-271, ARG-261, and PHE-264. Moreover, hydrogen bonds with ARG-265 and a salt bridge with ARG-261 enhanced the structural stability of the complex composition and the improved electrostatic complementarity (**Figure 6B**). These molecular docking results suggest that ZGSII can form stable interactions with both transcription factors. To further validate complex stability, 100-ns molecular dynamics simulations were performed in this study, confirming the robustness of the predicted binding modes over time.

The dynamic behavior of the complexes was evaluated using standard biophysical metrics, including RMSD, RMSF, Rg, hydrogen bonds, and SASA. In the C/EBPϵ-ZGSII complex, RMSD exhibited significant initial fluctuation followed by stabilization in the later stages of the simulation, indicative of conformational plasticity and adaptive binding characteristics. Conversely, the SPI1-ZGSII complex exhibited constrained fluctuations, indicating a rigid binding mode (**Figure 6C, D**). Although the binding modes of two ligands differed, their binding states remained stable throughout the simulations. Another key molecular dynamics metric, RMSF, revealed that while the flexible loop region of C/EBPϵ displayed moderate fluctuations, its ligand-binding site remained structurally stable. In contrast, SPI1 showed reduced residue mobility across the entire protein, consistent with its rigid scaffold structure (**Figure 6E**). Rg analysis revealed distinct structural dynamics: the C/EBPϵ-ZGSII complex exhibited gradual compaction throughout the simulation, indicating structural tightening around the ligand. In contrast, the Rg value of SPI1-ZGSII complex remained largely unchanged, suggesting pre-organized structural features that support rigid binding (**Figure 6F**). Hydrogen bond analysis revealed that the C/EBPϵ complex maintained more stable and persistent interactions, indicating a dynamically stable binding interface (**Figure 6G**). SASA calculations showed a slight decrease for C/EBPϵ, consistent with reduced solvent exposure and shielding of the binding site, whereas the SASA of SPI1 remained constant, in agreement with the structural rigidity of its binding pocket and unaltered solvent accessibility. (**Figure 6H**).

To better quantify the binding affinity of ZGSII for the two transcription factors, molecular mechanics-generalized born surface area (MM-GBSA) calculations were performed based on the MD simulation trajectories. The calculated binding free energies of -29.94 ± 5.40 kcal/mol for the C/EBPϵ-ZGSII complex and -14.12 ± 1.16 kcal/mol for the SPI1-ZGSII complex indicate strong binding interactions, with ZGSII exhibiting higher binding affinity toward C/EBPϵ (**Table 1**). Free energy landscape (FEL) analysis revealed a single deep energy basin of the C/EBPϵ complex, indicative of high conformational stability. In contrast, the SPI1-ZGSII complex exhibited a more dispersed FEL distribution with several shallow minima, indicative of a greater conformational variability and reduced stability (**Figure 6I**). Energy decomposition analysis revealed that the binding interactions are predominantly driven by electrostatic and van der Waals forces, as well as the nonpolar solvation contributions. In the C/EBPϵ-ZGSII complex, ARG261 and ARG265 were the primary contributions to binding energy, whereas hydrophobic residues such as isoleucine and leucine made moderate contributions to complex stabilization. In SPI1-ZGSII complex, LYS247, LEU248, and SER203 were the most significant energy contributors, suggesting that the complex is stabilized through a combination of hydrogen bonding and hydrophobic interactions (**Figure 6J**).

**Table 1:** Binding free energies and energy components predicted by MM/GBSA (kcal/mol).

System name	C/EBPϵ-ZGSII	SPI1-ZGSII
Δ*E*_vdw_	-30.01± 7.33	-14.52± 4.33
Δ*E*_elec_	-80.00 ± 27.38	-355.35 ± 15.61
ΔG_GB_	84.05 ± 23.39	358.12 ± 13.00
ΔG_SA_	-3.97± 0.78	-2.37± 0.44
ΔG_bind_	-29.94± 5.40	-14.12± 1.16

ΔE_vdW_: van der Waals energy.

ΔE_elec_: electrostatic energy.

ΔG_GB_: electrostatic contribution to solvation.

ΔG_SA_: non-polar contribution to solvation.

ΔG_bind_: binding free energy.

The above computational modeling suggests potential interaction of ZGSII with SPI1 and C/EBPϵ. To further validate the cellular level effects of ZGSII on the expression of these transcription factors, this study measured SPI1 and C/EBPϵ protein levels. Results showed that, compared with the control group, the expression of SPI1 and C/EBPϵ was significantly reduced in the CY model group; however, ZGSII intervention effectively upregulated the protein expression levels of both transcription factors (**Figures 6K, L**).

### 3.7 ZGSII induces differentiation and suppresses proliferation in NB4 cells

**Figure 7:**
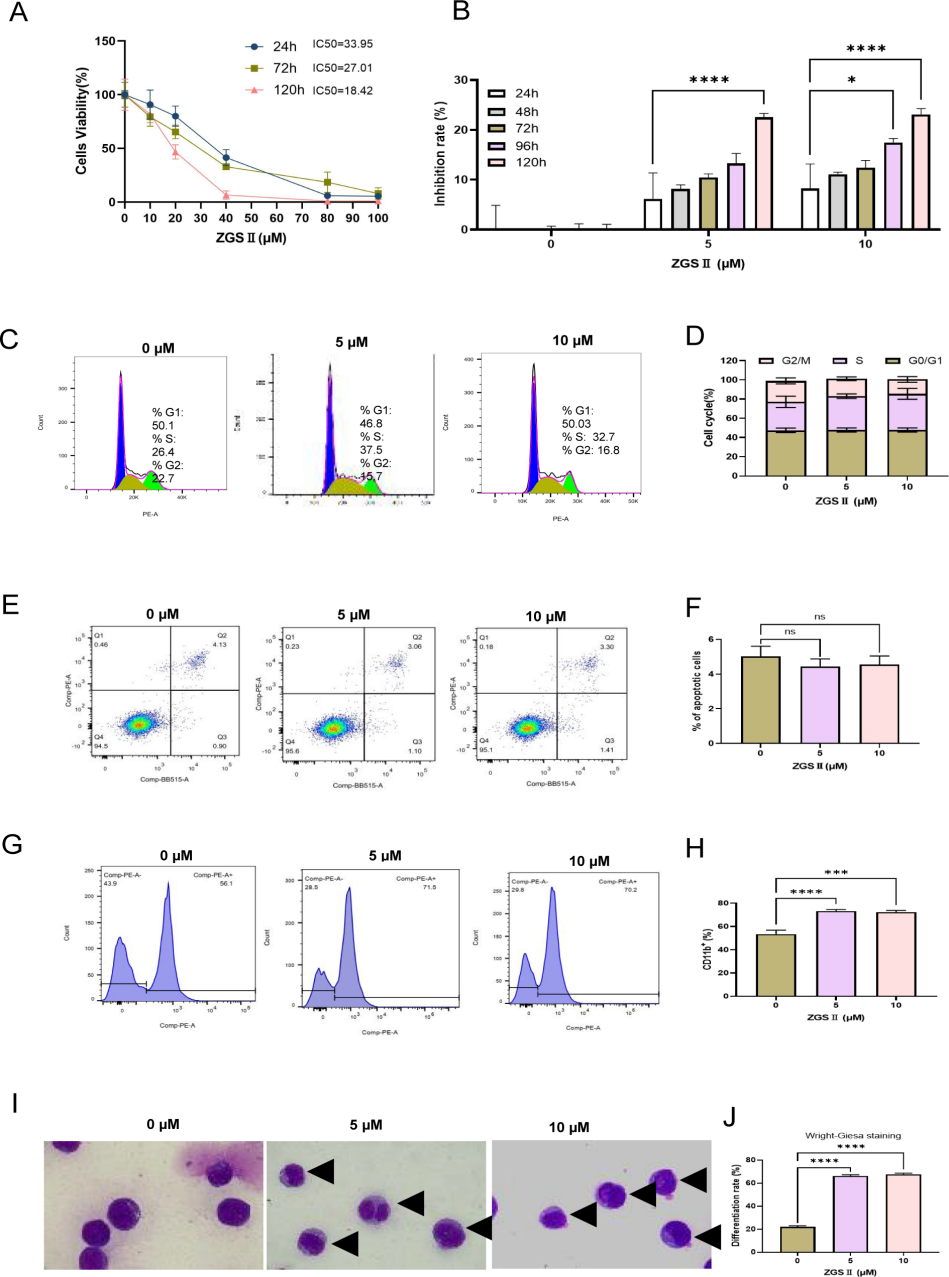
Ziyuglycoside II induces differentiation without cell cycle arrest or apoptosis in NB4 cells. **(A, B)** Dose- and time-dependent antiproliferative effects of ZGSII (0-100 μM, 24–120 h) assessed by CCK-8 assay. **(C-F)** Flow cytometric analysis demonstrating no significant changes in **(C, D)** cell cycle progression (propidium iodide staining) or **(E, F)** apoptosis (Annexin V/PI assay) following 120 h treatment at differentiation-inducing concentrations (5-10 μM). **(G-J)** Concentration-dependent neutrophilic differentiation evidenced by: **(G, H)** increased CD11b^+^ cell population (flow cytometry) and **(I, J)** characteristic morphological maturation in Wright-Giemsa stained NB4 cells (arrows indicate segmented nuclei). Data represent mean ± SD (n=3 independent experiments). *P < 0.05, ****P < 0.0001 versus vehicle control.

The human promyelocytic leukemia cell line NB4 is a well-established model for studying neutrophil differentiation, in which growth arrest serves as a key phenotypic marker (34, 35). To investigate the effects of ZGSII, NB4 cells were treated with increasing concentrations (0-100 μM) for 24–120 hours. CCK-8 assays revealed a dose-dependent suppression of cell growth (**Figures 7A, B**), with IC_50_ values decreasing from 33.95 μM at 24 h to 27.01 μM at 72 h, and further to 18.42 μM by 120 h. After 120 h of treatment, 10 μM ZGSII resulted in 23.11% growth inhibition. Flow cytometric analysis showed no significant alterations in cell cycle profile or apoptosis at concentrations of 5-10 μM after 120 h treatment (**Figures 7C–F**). In contrast, both flow cytometric and Wright-Giemsa staining confirmed concentration-dependent induced differentiation at these concentrations, accompanied by characteristic morphological maturation (**Figures 7G–J**).

### 3.8 ZGSII effectively enhances neutrophil function and confers a survival advantage in CIN

**Figure 8:**
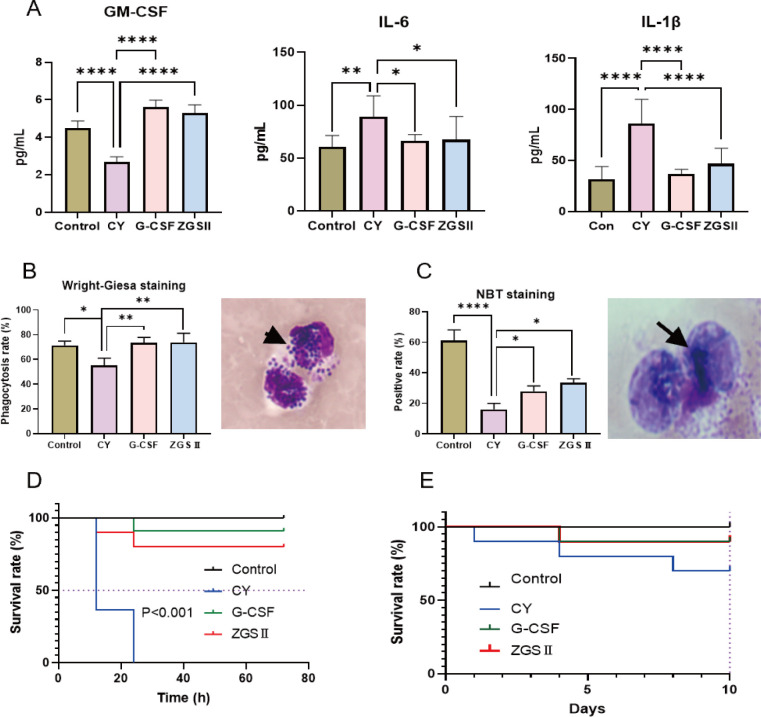
Ziyuglycoside II enhances neutrophil function and improves survival in bacteremic CIN mice. **(A)** Serum GM-CSF, IL-6, and TNF-α levels in BM cells from Con, CY, and ZGSII groups (n=5). **(B)** Representative Wright-Giemsa-stained micrographs (arrows indicate bacteria internalization), and quantified phagocytic index. **(C)** NBT reduction assay: Formazan deposition micrographs and NBT^+^ neutrophil percentage. **(D)** Kaplan-Meier survival curves of CIN mice challenged with high-dose *S. aureus* (5×10^7^ CFU, *i.p*.) monitored for 72 h (*P* < 0.001, log-rank test) (n=10/group). **(E)** Survival kinetics following low-dose *S. aureus* infection (5×10^6^ CFU, *i.p.*) over 10 days (n=10/group). *P < 0.05, **P < 0.01, ****P < 0.0001.

Furthermore, serum levels of the cytokines GM-CSF, IL-6, and TNF-α were measured using ELISA. The results showed that CY treatment significantly reduced serum GM-CSF levels, while markedly increasing the levels of the pro-inflammatory cytokines IL-6 and TNF-α. These alterations were notably reversed by G-CSF or ZGSII administration (**Figure 8A**). Functional assessments demonstrated enhanced neutrophil effector functions following ZGSII treatment: phagocytic capacity approached normal levels compared to the CY controls (*P* < 0.01; **Figure 8B**), while reactive oxygen species (ROS) production increased by 1.8-fold, as measured by the nitroblue tetrazolium (NBT) reduction assay (*P* < 0.05; **Figure 8C**).

This functional restoration correlated with improved survival in *Staphylococcus aureus*-challenged CIN mice. To evaluate protective efficacy, animals were infected intraperitoneally with high-dose (5×10^7^ CFU) or low-dose (5×10^6^ CFU) *S. aureus* on day 4 post-induction. In the high-dose infection group, median survival was reduced to 12 h in CY controls, whereas ZGSII significantly extended survival beyond this threshold (*P* < 0.001, log-rank test; **Figure 8D**). Following low-dose *S. aureus* challenge, only 70% of untreated mice in the CY group survived to day 10, compared to 90% survival in both G-CSF- and ZGSII-treated groups over the entire observation period (**Figure 8E**). These findings indicate that ZGSII confers significant survival benefits in bacteremic neutropenic hosts.

## 4 Discussion

Chemotherapy-induced neutropenia (CIN) is a major clinical complication that leads to treatment delay and risk of infection. Our study demonstrates that ZGSII effectively against CIN and preserves bone marrow homeostasis by promoting the neutrophil reconstitution through transcription regulation of key hematopoietic programs, while restoring neutrophil functions and enhances survival in bacteremic hosts.

ZGSII markedly replenished peripheral neutrophil populations and attenuated the bone marrow hypocellularity in the cyclophosphamide induced neutropenia model (**Figures 1A–F**), consistent with its previously reported hematopoietic protective properties. Notably, ZGSII enhanced neutrophil recovery without inducing excessive marrow mobilization, implying a balanced restoration of hematopoiesis and avoiding the risks of rapid progenitor cell depletion associated with G-CSF therapy (**Figures 2A–C**). In this study, a mild effect on monocytes was observed, whereas lymphocytes were not evidently affected by ZGSII. Immune infiltration analysis indicated that certain immune cell subsets, such as Treg cells and Th1 cells, were influenced to some extent. The impact on other immune cells, particularly those within the tumor microenvironment, warrants further in-depth investigation (36, 37). Of course, these immune cell proportion estimates derived from CIBERSORT should be interpreted as relative trends rather than absolute quantification.

RNA-seq analysis revealed that the ZGSII administration was associated with a substantial reversal of CY-induced transcriptional alterations, particularly in genes clustered within pathways related to leukocytes migration, myeloid cell activation, and regulation of the inflammatory response. This change was not only quantitative but qualitatively significant, as evidenced by the enrichment of several KEGG pathways, such as leukocyte transendothelial migration, cytokine-cytokine receptor interaction and hematopoietic cell lineage, pathways essential for proper neutrophil development and function.

Another critical process is the regulation of the bone marrow immune microenvironment. ZGSII suppressed CY-induced changes in immune cell infiltration, thereby creating a more favorable niche for neutrophil development. This modulation of the microenvironment, combined with direct transcriptional regulation of myeloid differentiation programs, positions ZGSII as a versatile therapeutic agent for neutropenia.

The overlap between DEGs and neutrophil subpopulation signatures from publicly available datasets identified 37 relevant genes, which showed increased neutrophil differentiation and maturity score following ZGSII treatment. A protein-protein interaction network constructed from these genes revealed a number of core regulators such as Ltf, Lcn2, Cxcr2, Mmp8/9 and S100a8/9 that collectively forms a functional network associated with neutrophil maturation, migration and antimicrobial activity (38–40). The bacterial iron binding protein Ltf (lactotransferrin) and the innate immune system protein Lcn2 (lipocalin 2) play essential in iron sequestration and host defense, while Cxcr2 serves as the primary chemokine receptor mediating neutrophil mobilization from bone marrow to peripheral tissues (41–44). Increased expression of Mmp8/9 and S100a8/9 further suggests enhanced neutrophil degranulation potential and pro-inflammatory signaling (45, 46), consistent with our functional assays demonstrating restored phagocytic capacity and ROS generation.

The mechanistic observations indicate that ZGSII mediates neutrophil differentiation by regulating key transcriptional regulators. Computational docking results revealed high binding affinities between ZGSII and both SPI1 (-7.073 kcal/mol) and C/EBPϵ (-7.235 kcal/mol), with molecular dynamics simulations provided detailed insight into the binding dynamics: C/EBPϵ/ZGSII complex exhibited high ligand fluctuation during early stages, which stabilized in late stages, conformational plasticity and adaptive binding behavior; in contrast, SPI1/ZGSII complex showed moderately restrained fluctuations, indicating a more rigid and stable interaction at the binding site. These findings were functionally validated by the observation that ZGSII treatment reversed the CY-induced suppression of both transcription factor protein expression.

SPI1 is a hematopoietic stem cell fate regulator and a master regulator of myeloid lineage commitment (47, 48), whereas C/EBPϵ regulates terminal granulocytic differentiation and secondary granules formation (49, 50). MD simulation results revealed that the SPI1/ZGSII complex exhibited a rigid binding mode, while the C/EBPϵ/ZGSII complex displayed conformational plasticity and adaptive binding, indicating active engagement of ZGSII with these two transcription factors through distinct mechanisms. This may involve activation of SPI1 by ZGSII, driving the differentiation of hematopoietic stem cells into the myeloid lineage, as well as induction of C/EBPϵ activation to promote granulocyte maturation. These transcription factors are coordinately regulated by ZGSII, suggesting a sophisticated mechanism underlying its pro-myelogenic regulatory activities. However, although molecular docking and MD simulations suggest stable interactions between ZGSII and SPI1 or C/EBPϵ, direct biochemical validation (e.g., SPR, CETSA, pull-down assays) will be required to confirm physical binding.

NB4 cells treated with ZGSII exhibited growth inhibition that was specifically associated with myeloid differentiation rather than general cellular proliferation. The restoration of transcriptional programs that drive neutrophil maturation likely underlies this functional recovery: ZGSII restored neutrophil phagocytic capacity and enhanced ROS production, as measured by NBT reduction assay, indicating full functional reconstitution beyond mere numerical recovery.

These immunomodulatory effects of ZGSII were further evident in cytokine regulation. CY significantly reduced serum GM-CSF levels and markedly increased pro-inflammatory cytokines such as IL-6 and TNF-a, which were substantially reversed by ZGSII. This cytokine profile modulation indicates that ZGSII not only enhances neutrophil production but also promotes a hematologically favorable microenvironment for neutrophil function, thereby reducing the inflammatory burden associated with chemotherapy-related neutropenia.

Above all, these cellular and molecular improvements translated into significant survival benefits in CIN mice infection with *S. aureus*. Animals treated with ZGSII exhibited markedly improved survival rates following both high-dose and low-dose bacterial inoculation, thereby confirming the functional relevance of the observed hematological recovery. This protective effect against Gram-positive pathogens holds substantial clinical relevance, particularly in neutropenic patients, where bacterial infections represent a leading cause of morbidity and mortality.

ZGSII appears to confer a distinct therapeutic advantage over conventional growth factor therapies, such as G-CSF, which primarily stimulate cellular proliferation. While G-CSF accelerates neutrophil proliferation, ZGSII enhances directional differentiation and functional maturation through transcriptional-regulation. Furthermore, its cytokine-modulating effects may help mitigate inflammatory complications commonly associated with chemotherapy.

Our present findings extend of our previous study by elucidating the establishes the transcriptional networks underlying the biological mechanisms through which ZGSII exerts its protective effects against CIN. The dual targeting of SPI1 and C/EBPϵ signaling represents a novel therapeutic strategy that may be beneficial compared to current growth factor-based approaches, which predominantly promote cellular proliferation rather than differentiation. These results may provide a preclinical proof for ZGSII as a therapeutic adjuvant or alternative treatment option for CIN.

Nevertheless, several limitations should be acknowledged. First of all, the current research findings indicate that the drug exhibits no significant toxicity in short-term. However, the potential for long-term toxicity, PK/PD studies or comparison with pegylated G-CSF requires further investigation. Moreover, the study primarily relied NB4 cell lines, which represent a well-established model of granulocytic differentiation. However, they may not fully recapitulate the biology of normal hematopoietic progenitor or faithfully model human hematopoiesis. These findings warrant validation in primary human hematopoietic stem and progenitor cells and in clinically relevant samples in future studies. Lastly, future studies using genetic or pharmacological inhibition of SPI1 and C/EBPϵ will be necessary to establish their causal role.

## 5 Conclusion

In conclusion, our results demonstrated that ZGSII significantly ameliorates chemotherapy-induced neutropenia by promoting neutrophil reconstitution through transcriptional regulation of SPI1 and C/EBPϵ. These findings provide a preclinical proof for ZGSII as a therapeutic agent to mitigate chemotherapy-induced hematological toxicities without compromising treatment efficacy.

## Data Availability

The datasets presented in this study can be found in online repositories. The names of the repository/repositories and accession number(s) can be found below: PRJNA1390471 (SRA).
