# Supplementary material for: Ziyuglycoside II ameliorates chemotherapy-induced neutropenia by promoting neutrophil differentiation and functional recovery via SPI1 and C/EBPϵ transcriptional regulation
Source: Front Immunol. 2026 Feb 26;17:1771161. doi: 10.3389/fimmu.2026.1771161 (PMC12979075; doi:10.3389/fimmu.2026.1771161)
Supplement: Supplementary file 3 [file Table3.docx]

Supplement Table 3. The sequences of RT-qPCR primers used for BM cells

| Gene | Sequence F (5′–3′) | Sequence R (5′–3′) |
| --- | --- | --- |
| Ltf | GTCTGCCATTGGCTTTGTGAGG | CCTTTGAGGCTATCACATCCTGC |
| Cxcr2 | TTCGCTGTCGTCCTTGTCT | CCTCCTTGGCTAAGAACTCCT |
| Fpr2 | GCAGTTGTGGCTTCCTTCTT | AACATAGAGCATCGGATTGAGAC |
| S100a8 | GAGCAACCTCATTGATGTCTACC | GTCACTATTGATGTCCAATTCTCTG |
| Itgb2l | GCTGGATGCTGTGGTTCAAG | CGCTGGTGTTCTGTCTGGTA |
| Anxa1 | GCAACCATCATTGACATTCTTACC | GGCACCACGGAGTTCATCT |
| 18SrRNA | ATACATGCCGACGGGCGCTG | CGGCTCGGGCCTGCTTTGAA |
